# Do improved structural surroundings reduce restrictive practices in psychiatry?

**DOI:** 10.1186/s13033-022-00562-7

**Published:** 2022-11-20

**Authors:** Astrid Harpøth, Harry Kennedy, Morten Deleuran Terkildsen, Bettina Nørremark, Anders Helles Carlsen, Lisbeth Uhrskov Sørensen

**Affiliations:** 1 Department of Forensic Psychiatry, Aarhus University Hospital Psychiatry, Skejby, Denmark; 2 Trinity College, Dublin University, Dublin, Ireland; 3 National Forensic Mental Health Service, Dundrum, Ireland; 4 DEFACTUM, Central Denmark Region, Aarhus, Denmark; 5 Department of Clinical Medicine, Faculty of Health, Aarhus University, Aarhus, Denmark

**Keywords:** Coercion, Structural safety, Psychiatry, Mechanical restraint, Restrictive practices

## Abstract

**Background and objectives:**

There is sparse evidence that modern hospital architecture designed to prevent violence and self-harm can prevent restrictive practices (RP). We examine if the use of RPs was reduced by the structural change of relocating a 170-year-old psychiatric university hospital (UH) in Central Denmark Region (CDR) to a new modern purpose-built university hospital.

**Methods:**

The dataset includes all admissions (N = 19.567) and RPs (N = 13.965) in the self-contained CDR one year before and after the relocation of the UH. We compare RPs at the UH a year prior to and after relocation on November 16th (November 2017, November 2019) with RPs at the other psychiatric hospitals (RH) in CDR. We applied linear regression analysis to assess the development in the monthly frequency of RPs pre- and post-relocation and examine underlying trends.

**Results:**

At UH, RPs performed decreased from 4073 to 2585 after relocation, whereas they remained stable (from 3676 to 3631) at RH. Mechanical restraint and involuntary acute medication were aligned at both UH and RH. Using linear regression analysis, we found an overall significant decrease in the use of all restrictive practices at UH with an inclination of -9.1 observations (95% CI − 12.0; − 6.3 p < 0.0001) per month throughout the two-year follow-up. However, the decrease did not deviate significantly from the already downward trend observed one year before relocation. Similar analyses performed for RH showed a stable use of coercion.

**Conclusion:**

The naturalistic features of the design preclude any definitive conclusion whether relocation to a new purpose-built psychiatric hospital decreased the RPs. However, we argue that improving the structural environment at the UH had a sustained effect on the already declining use of RPs, particularly mechanical restraint and involuntary acute medication.

## Introduction

Psychiatric inpatient aggression may lead to the prescription of restrictive practices (RP) such as seclusion, restraint or involuntary acute medication [1]. The use of RPs in psychiatry is controversial but considered necessary to prevent violent behaviour toward others in hospital wards and prevent self-harm and suicide. It is widely agreed that RPs should be minimised as patients report primarily adverse effects of being subjected to such measures [2]. RPs have been found to cause physical and psychological harm [3], lead to violence, and damage the therapeutic alliance, widely acknowledged as essential to achieving patient recovery [4, 5]. Conversely, violence prevention in psychiatric wards is essential to maintain a therapeutically safe environment and protect other patients and staff [1]. RPs may be prevented and reduced by optimising procedural, relational-dynamic, and structural factors [6].

The relational-dynamic and procedural factors and their influence on RP have been extensively researched [7]. Both improvements in staff training, guideline implementation, and systematic changes in the dynamic environment within the wards reduce the use of RPs [8, 9].

Contrastingly, the effect of structural surroundings on the use of RP in psychiatry has received less attention [10].

Ulrich et al. have proposed a conceptual model for how psychiatric ward design may affect aggression and, thus, ultimately, RP. The fundamental premise is that psychiatric inpatients experience stressors that foster and trigger aggression. The physical ward environment can influence these stressors. Thus, a well-planned evidence-grounded ward design may minimize environmental-related stress by reducing crowding and noise and providing positive distractions such as gardens accessible to patients, windows with nature views and increased exposure to daylight in the wards [11]. In line with this, other models have suggested that high quality of the structural surroundings, such as the conditions and cleanliness of the buildings and the decor, may reduce conflicts in psychiatric wards [12].

However, even though current literature has affirmed the physical environment's importance in supporting better mental health services outcomes, more rigorous research is needed to establish the link between structural surroundings and RP [7, 10, 11, 13].

Older psychiatric buildings from the asylum era may be inadequate to support treatment as usual and prevent RP [14]. However, only a few studies have examined how modern hospital architecture designed to prevent violence and self-harm and support de-escalation affects the use of RP as evidence concerning architectural design features is frequently published in the grey literature [13].

A unique opportunity to examine the effect of structural surroundings on RP was presented when the old psychiatric University Hospital (UH) in Aarhus, dating back to 1852, relocated to a new modern purpose-built psychiatric hospital in November 2018, thus, creating a quasi-experimental situation. This study examines if these improved structural surroundings decreased restrictive practices in psychiatric inpatient units.

## Method

### Setting

Denmark consists of five self-contained regions, one of which is the Central Denmark Region (CDR), with approximately 1.3 million inhabitants, 23% of the Danish population [15]. The psychiatric hospitals in CDR consist of one university hospital (UH) and four regional hospitals (RH). All hospitals are considered “one organisational unit” governed by the same leadership. Following the Danish Mental Health Act, the overall national regulations and those specific to psychiatry, the UH offer specialised treatment for the most complicated and severely ill patients in CDR [16, 17].

Aarhus University, the Faculty of Health and the Central Denmark Region have a long-standing formalized collaboration centred on research and education [18]. For CDR, the collaboration within psychiatry is primarily centred and organized from Aarhus University Hospital Psychiatry (in this paper, UH), as the psychiatric professors and the majority of associate professors are affiliated with Aarhus University Hospital Psychiatry. The UH employed 1476 full-time staff members on the 15^th^ of November 2018; the corresponding number at the four regional hospitals was 1163 (ranging from 171 to 493). There is a considerable shortage of psychiatrists in Denmark, particular at the RHs throughout Denmark; this is also and in particular the case for CDR [19].

### Mental Health Act concerning restrictive practices

The use of restrictive practices in psychiatry is regulated by the Mental Health Act (MHA), which states that RP’s can only be applied to patients admitted to a psychiatric hospital and must be prescribed by psychiatrists. The MHA regulates; involuntary admissions and detentions, involuntary treatments (acute and prolonged medication, ECT, nourishment), restraint (mechanical or by staff), surveillance by staff, and involuntary treatment of life-threatening somatic conditions [20, 21].

In 2014 the Government and the Regions (hospital owners) decreed that the overall level of restrictive practices described in the MHA should be reduced, and the number of individuals prescribed mechanical restraint should be halved by 2020. To achieve this goal, all five Regions implemented Safewards [22].

### Legislation concerning restrictive practices

The Danish MHA defines coercion as:” measures for which there is no informed consent” [21].

Informed consent is provided based on adequate information from the healthcare professional about the state of health, treatment options, the risk of complications and side effects. The consequences of non-treatment must also be informed. The consent is only valid if it has been given voluntarily. Thus, consent is invalid if it is given under unacceptable pressure, coercion or concealment of the truth [23]. In mechanical restraint, a belt is applied around the waist and sometimes straps for the extremities to fix the patient to a bed. Mechanical restraint must be accompanied by 1:1-surveillance. Involuntary acute medication is administering acute medication (oral, intramuscular, or intravenous) without informed consent. The coercion to administer the medication may be physical or psychological.

### Study design

The study is designed as a naturalistic retrospective quasi-experimental pre—and post-study that includes admissions for all individual patients (N = 7.566) admitted to all the psychiatric hospitals (RHs and UH) in CDR from the 15th of November 2017 to the 16th of November 2019. We compare the use of RP stratified by RH and UH one year before and one year after the relocation of the UH, which took place in mid-November 2018. We set the relocation date to the 16th of November 2018. The RHs did not relocate; furthermore, the internal organizational structure and training on restrictive practices remained the same. The RHs thus serve as a comparison group for the "relocation" patient population at the UH.

### Total dataset

The data included a total of 19.567 *admissions* from CDR during the study period. The admitted patients at the RH were older, and a higher proportion were females (Table 1).

**Table 1:** All *admissions* at the University and Regional Psychiatric Hospitals in Central Denmark Region before and after the relocation of the University Hospital on November 16th 2018. Demographic, diagnostic and clinical characteristics of admissions

	University hospital		p-value	Regional hospitals		p-value
	Pre-relocation period^1^	Post-relocation period^2^		Pre-relocation period^1^	Post-relocation period^2^	
Demographics, all admissions						
Age, years, mean (SD)	38 (17.4)	37 (16.4)	0.0238	40 (17.9)	40 (16.8)	0.074
Sex, female, n (%)	1902 (52)	2038 (55)	0.016	3842 (54)	3739 (58)	< 0.0001
ICD- 10 primary diagnosis, all admissions	n (%)	n (%)		n (%)	n (%)	
F0 Organic mental disorders, n (%)	98 (3)	53 (2)		181 (3)	175 (3)	
F1 Psychoactive substance use disorder, n (%)	225 (6)	156 (4)		475 (8)	555 (8)	
F2 Psychotic disorders, n (%)	1149 (32)	1228 (35)		1643 (28)	1799 (28)	
F3 Mood disorders, n (%)	938 (26)	834 (24)		1147 (20)	1393 (22)	
F4 Anxiety disorders, n (%)	436 (12)	374 (11)		984 (17)	968 (15)	
F5 Eating disorders, n (%)	136 (4)	113 (3)		53 (1)	40 (1)	
F6 Personality disorders, n (%)	364 (10)	450 (13)		586 (10)	689 (11)	
F7 Mental retardation, n (%)	42 (1)	46 (1)		116 (2)	75 (1)	
F8 Disorders of psychological development, n (%)	81 (2)	73 (2)		207 (4)	285 (4)	
F9 Behavioral disorders in childhood, n (%)	60 (1)	67 (2)		126 (2)	145 (2)	
Other diagnoses, n (%)	124 (3)	125 (4)		398 (7)	360 (6)	
Total number of admissions, N (%)	3653 (100)	3519		5916	6479	
Clinical characteristics, all admissions, N						
Readmission within 30 days, n (%)	943 (26)	1031 (30)	0.001	1829 (31)	2346 (36)	< 0.0001
Any diagnosis of substance use disorder, n (%)	668 (18)	582 (17)	0.051	1039 (18)	1110 (17)	0.527
Forensic psychiatric admissions, n (%)	540 (15)	658 (19)	< 0.0001	754 (13)	832 (13)	0.873

^1^November 15th 2017 to November 15th 2018

^2^16th November 2018 to November 16th 2019

### Data and outcome measures

We drew data from the CDR Business Intelligence (BI) portal, which contains data from the electronic patient record and the national register for restrictive practices in psychiatry. Our dataset constitutes a complete sample during follow-up of all admitted patients and corresponding RPs at CDR psychiatric hospitals. The outcomes of RPs were; total numbers of RPs, mechanical restraint, and involuntary acute medication.

To evaluate changes that may affect the use of RP during follow-up, we included data on: patient age and gender, hospital of admission (UH versus RH), length of stay, readmission proportion, date of admission, and forensic status (forensic patient versus not). For each admission, we selected the highest-ranking diagnosis in the International Classification of Diseases, tenth revision (ICD-10) as the primary diagnosis, excepting F1 diagnoses [24]. F1 diagnoses were only registered as a primary diagnosis if this was the only diagnosis. Furthermore, we constructed a variable, substance use disorder (SUD), if any F1 diagnosis was registered during the study period.

For all RPs regulated according to the Mental Health Act, we included; type, date, and duration. The Act was revised twice during follow-up, and one type of RP was removed with the revision May 2019 [25] due to lack of use (< 10 incidents per year in CDR). Seclusion and time-outs are not permitted according to the MHA.

### Relocation; the new hospital

The relocation of the UH took place in mid-November 2018. The old UH was inaugurated in 1852 outside the centre of Aarhus [26]. The new UH is purpose-built on Aarhus's somatic university hospital campus. The new UH was designed to improve; 1) security for patients, staff and the public, 2) efficiency of in and outpatient treatment, 3) somatic treatment for psychiatric patients by closer proximity to the somatic hospital and 4) social well-being [27].

A large part of the old UH buildings maintained the outwards appearance of an asylum. Contrastingly, the new UH resembles the adjacent somatic hospital. The new UH has 257 beds, a reduction of 7 beds compared to the old UH. The number of beds at the RH was 253 throughout the study period. Security for staff, patients, and community and patient privacy are prioritised. Both the new and old UH features mostly single rooms, whereas the new UH patient rooms all have large ensuite bathrooms. The wards at the new UH have airlocks, wider halls, and anti-suicidal features avoiding ligature points and hiding cables. At the old UH, fencing provided perimeter control for shared gardens, and access to fresh air was limited. The new UH has structurally integrated courtyards, providing patients unlimited access to round-the-clock outdoor areas. At the new UH, four 16-bed wards were merged into two 26-bed wards. The m^2^ per patient in general psychiatry increased from 55 m^2^at the old UH to 67 m^2^ at the new hospital, patient rooms increased from 15.5 m^2^ to 18.6 m^2^, and the area for activity rooms increased from 48,5 to 60.6 m^2^.

During the study period, the number of admissions declined at the UH and increased at the RHs. The decline in admissions was, among others, probably caused by the decrease in beds at UH; furthermore, the catchment area for UH decreased at the new UH while a similar increase took place at RHs. Finally, patients could refer themselves to the psychiatric emergency room at the old UH, whereas referral from a physician is required at the new UH (Table 1).

### Statistical analysis

We used descriptive statistics to quantify the characteristics of the study population, use of RP, and admissions. We used the chi-square test to examine the following categorical: sex, type of coercive measure, diagnosis, forensic psychiatric patients, and diagnosis of substance use disorders (ICD-10, F1). We used the Mann–Whitney U test on the numerical variables: age, duration of manual restraint, duration of mechanical restraint, and length of admission at the time of the coercive measure.

We applied linear regression analysis to assess change or trend in the monthly frequency of RP. The use of linear regression enabled us to examine if any changes in the underlying trend in the use of RPs could be attributed to the relocation of the UH. Linear regression models were fitted to examine the effect of relocation, stratified on location, allowing for different slopes pre and post-relocation and with and without continuity at the point of relocation. These models were compared using likelihood-ratio tests to a model that enforced the same linear slope throughout the study period.

Data management and analyses were conducted using Stata 16 software [28].

### Ethics and data security

The study was registered at the Central Denmark Region Research Database (file number: 1-16-02-9-20) and approved by the Danish Patient Safety Authority (file number:31-1521-146).

## Results

### Changes in admission patterns during the study period

The number of admissions fell (from 3.653 to 3.519) at the UH during the study period, whereas it increased at RH (5.916 to 6.479). During the study period, the main change in admission pattern was a significant increase at both UH and RH in the proportion of readmissions and females admitted. The proportion of admissions where the patient had *any* substance use disorder diagnosis did not increase at either UH or RH. The overall diagnostic distribution remained fairly stable during the study period at both RH and UH (Table 1).

### Restrictive practices

A total of 13.965 RPs were prescribed for 2.114 individual patients in CDR during the observation period. At UH, the number of RPs performed decreased from 4.073 before relocation to 2.585 after relocation, whereas it remained stable (from 3.676 to 3.631) at RH (Table 2). Throughout the study period, the overall use of RPs was lower at the RH compared to the UH when measured as RPs per admission: with 0.9 RP per admission at UH versus 0.6 at the RHs.

**Table 2:** All restrictive practices at the University and Regional Psychiatric Hospitals Central Denmark Region before and after relocation of the University hospital November 16th, 2018

	University hospital		p-value	Regional hospitals		p-value
	Pre-relocation^1^	Post-relocation^2^		Pre-relocation^1^	Post-relocation^2^	
	n (%)	n (%)		n (%)	n (%)	
Restrictive practices						
Involuntary admission	454 (11)	403 (16)	< 0.0001	657 (18)	771 (21)	< 0.0001
Involuntary detention	284 (7)	204 (8)	0.161	317 (9)	320 (9)	0.774
Locking of doors for an individual patient	176 (4)	99 (4)	0.326	262 (7)	222 (6)	0.082
Mechanical restraint	788 (19)	373 (14)	< 0.0001	360 (10)	345 (10)	0.673
Straps (wrists and ankles)	555 (14)	295 (11)	0.008	246 (7)	272 (8)	0.183
Manual restraint	373 (9)	367 (14)	< 0.0001	427 (12)	355 (10)	0.011
Involuntary personal shielding for more than 24 h	8 (0)	7 (0)	0.533	4 (0)	6 (0)	0.514
Involuntary acute medication	1202 (30)	628 (24)	< 0.0001	1119 (30)	981 (27)	0.001
Involuntary treatment	82 (2)	54 (2)	0.831	118 (3)	110 (3)	0.657
Involuntary electroconvulsive therapy	15 (0)	8 (0)	0.690	14 (0)	30 (1)	0.014
Involuntary nutrition	20 (1)	45 (2)	< 0.0001	6 (0)	18 (1)	0.013
Involuntary treatment of a somatic disorder	94 (2)	86 (3)	0.012	138 (4)	184 (5)	0.006
Other	22 (1)	16 (1)	0.677	8 (0)	17 (1)	0.067
Total number of restrictive practices (N)	4073	2585		3676	3631	

^1^November 15th 2017, to November 15th 2018

^2^November 16th 2018, to November 16th 2019

The proportion of involuntary admissions (involuntary admission/all admissions) remained stable after the relocation (UH: 12% before and after; RH: 11% before and 12% after).

The proportion of restrictive practices performed during the first 24 h of admission at UH increased from 33% (1332/4073) pre-location to 42% (1096/2585) post relocation (chi^2^ p < 0.0001), at RH it increased from 44% (1630/3676) to 52% (1885/3631) (chi^2^, p < 0.0001).

At UH, the median length of mechanical restraint increased from 5.8 h (IQI 2.0—15.1) to 6.8 h (IQI 2.5—18.8) post-relocation (two-sample Wilcoxon rank-sum test, p = 0.011). Correspondingly at RH, the median length of mechanical restraint increased from 7.8 h (IQI 2.4—16.1) before to 9.2 h (IQI 3.5—18.3) two-sample Wilcoxon rank-sum test (p = 0.0425).

**Fig. 1:**
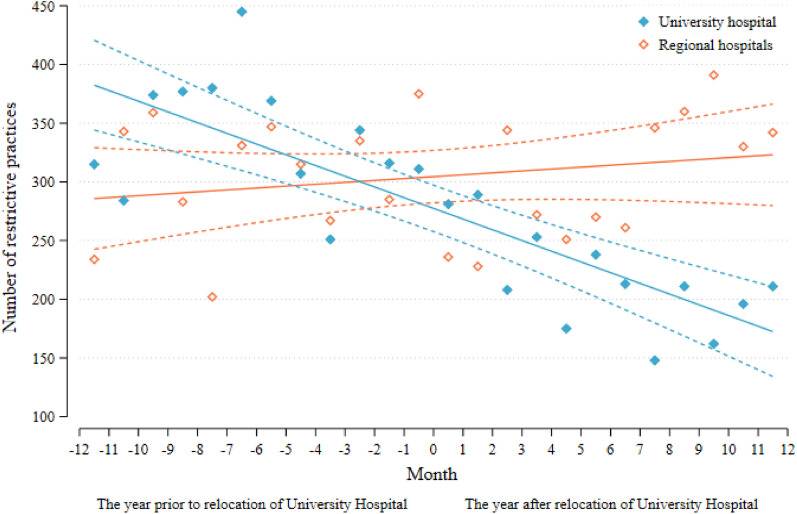
Restrictive practices at the University and Regional Psychiatric Hospitals Central Denmark Region by pre-and post-relocation of the University hospital November 16^th^ 2018

Using linear regression analysis, we found an overall significant decrease in the use of all restrictive practices at UH with an inclination of -9.1 observations (95% CI -12.0;-6.3 p < 0.0001) per month throughout the two-year follow-up. However, the decrease post-relocation did not deviate significantly from the already downward trend observed one year before relocation. Similar analyses performed for RH showed a stable use of coercion (Fig. 1). Thus, we did not find evidence to support the hypothesis of significantly different slopes pre- and post-relocation for the total use of coercive measures in either of the two locations.

### Use of mechanical restraint and involuntary acute medication

**Fig. 2:**
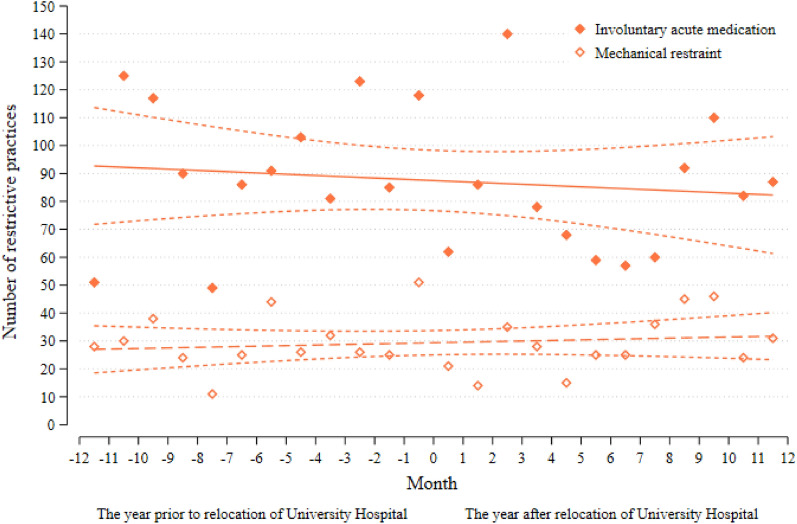
The use of involuntary acute medication and mechanical restraint at the Regional Hospitals in Central Denmark Region 2017–2019 before and after relocation of the University Hospital November 16th 2018

**Fig. 3:**
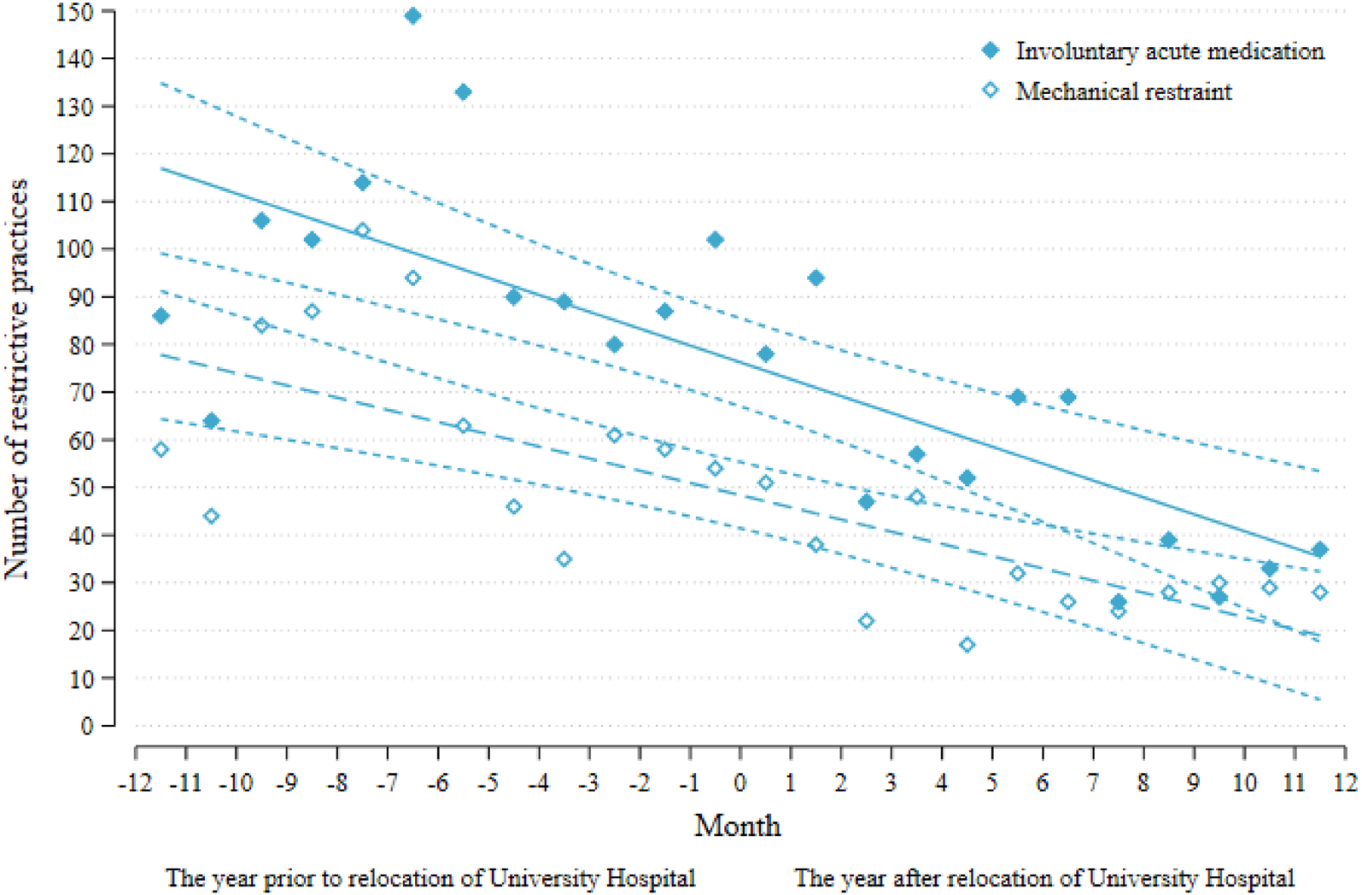
The use of involuntary acute medication and mechanical restraint at the University Hospital the University in Central Denmark Region 2017–2019 before and after relocation of the University Hospital November 16th 2018

For both UH and RH, mechanical restraint and involuntary acute medication were aligned (Figs. 2 and 3), and the post-relocation slopes did not deviate significantly from the observed trend before the location.

Overall, the *numbers* for mechanical restraint and involuntary acute medication numbers at the UH were nearly halved during the study period, whereas the *numbers* for manual restraint remained fairly stable (from n = 373 to n = 367) (Table 2).

The numbers for mechanical restraint, manual restraint, and involuntary acute sedation at the RH decreased (Table 2).

### Use of restrictive practices in somatic wards

Linear regression analyses showed a stable use of mechanical restraint on patients stationed in the somatic wards at UH, whereas RH had a minor insignificant increase (data not shown).

## Discussion

### Summary of results

We conducted a register-based retrospective quasi-experimental pre—and post-study with a relocation group (UH) and a comparison group (RH) to examine the effect of structural changes on the use of restrictive practices in Denmark from 2017 to 2019. Our main findings were as follows; first, the actual number of RPs decreased at the relocation hospital (UH) compared to the comparison hospitals (RHs); however, this decrease was not significantly different from the expected underlying trend. Secondly, mechanical restraint and acute involuntary medication were aligned, and there was no indication that a decrease in mechanical restraint was substituted with increased use of involuntary acute medication. To some extent, mechanical restraint was substituted with manual restraint. Finally, the use of RPs in the somatic wards (involuntary treatment of somatic disorder) remained fairly stable at the UH while it increased slightly at the RHs.

### The effect of improved structural surroundings on the total use of RP

Our results should not be interpreted to indicate that improvement in structural surroundings had no effect. Even though our results were not statistically significant in the linear regression analysis, we argue that the improved structural surroundings reinforced or maintained the declining use of RPs observed at the UH. Firstly, organizational changes are often marred by considerable challenges, especially in their early stages, and often, changes fail to meet the stated goals [29]. This may, in part, be attributed to the impact of change in work practices and environments challenging humans’ fundamental need for stability [30]. Moreover, changes in work practices and environment have been shown to reduce organizational commitment and productivity and increase work-related stress [31]. Our study did not measure these parameters, nor did we include any data concerning staff turnover. However, the relocation caused considerable organisational upheaval and staff turnover. The staff turnover mainly consisted of staff changing from one position (ward/outpatient clinic) at the old UH to another at the new UH, which meant that the input of "new staff" was very low. Thus, the main change due to the relocation was the disruption of multidisciplinary teams and groups, which could be argued to affect clinical performance negatively [32]. On the other hand, it might be hypothesized that the continued decrease of restrictive practices during relocation might be linked to robust organizational resilience [33].

These disruptions posed a greater risk of increasing RPs rather than decreasing them, indicating that the changed structural surroundings here may have played a key role in continuing the downward trend. Secondly, RH and UH are part of the same organisation and are governed by identical procedural and dynamical factors. Thus, the main differences between RH and UH during the study were the structural change of relocation at UH.

Thirdly, one of the aims of the new UH hospital was to increase safety and security by reducing violence. Thus, the new UH was designed to reduce crowding, provide private space, and promote well-being by using natural light, all of which have been suggested as instrumental when reducing violence in psychiatric wards [34, 35]. Rohe et al., who examined the effect of a hospital relocation on mechanical restraint in a before and after study without a control group, found a significant decrease in the number of patients subjected to mechanical restraint after relocation and attributed it to the following: the considerable structural improvements (i.e., single rooms, more light) in the new hospital, increased training of staff in de-escalation measures and changes in legislation during the study period [36]. Similar results and deliberations have been found by others who studied the effects of relocations in psychiatry [11, 37]. Furthermore, a recent rapid systematic review found "preliminary evidence that physical design features of mental health facilities can reduce the use of seclusion and physical restraint” [13].

The overall RPs per patient subjected to RPs admission were higher at the UH compared to RH. According to the Danish Mental Health Act, RPs must be prescribed by a psychiatrist. The number of MDs per patient was considerably higher at the UH than at the RH. In line with Roemer's law which states that there is a relationship between hospital bed availability and inpatient hospitalization rates, our study might indicate an iatrogenic impact of MD on restrictive practices as these can only be prescribed by an MD [38]. According to hospital planning, the UH is regulated to treat the most treatment-resistant and complex patients from the entire CDR. However, our data do not allow us to examine and describe to what extent this actually affects the patient population at the UH, and, ultimately, the use of RPs.

### Substituting restrictive practices

Restrictive practices are often divided into four categories: therapy by use of RP (prescription of medication), use RP without primary purpose (chemical restraint), separation (e.g., seclusion), and mechanical restriction (e.g., restraint by belts and straps). Tradition, legislation, and culture seem to determine preferences when using restrictive practices [39, 40]. In line with a Danish study examining the effect of Safewards on RP [41], we found that the prescription of involuntary medication and mechanical restraint was aligned with a simultaneous decrease in both mechanical restraint and involuntary acute medication. Similarly, a Danish study found that a reduction in the use of mechanical restraint did not increase the overall use of antipsychotics and benzodiazepines [42]. Contrastingly, a nationwide Dutch study found that even though seclusion decreased significantly, “forced medication” increased; however, the pattern was not uniform as the rates varied between hospitals [43]. Our study does not include any data on other compensating reactions, such as increasing use of informal coercion or sedatives [44]. Still, we have no reason to believe these should differ at RH and UH.

The use of manual restraints remained stable at the UH, while the use of mechanical restraint and acute involuntary medication decreased; thus, we did not find that “*a ban of one kind of measure seems to lead to an increase of others”* [39]. It has been put forward that the nursing staff finds manual restraint challenging to perform, confrontational, and negatively affects the relationship with the patient [45]. To prevent protracted manual restraints, the Danish National Board of Health issued a maximum length of manual restraint of 30 min in 2020 [46]. The least restrictive and most effective RP may be decided by individual patient preferences, legislation, culture, and the context of action. This lack of clarity can be attributed to a lack of sound scientific evidence, especially a lack of a system for description and measurement. A way forward has been described with the Dundrum Restriction and Intrusion Liberty Ladders (DRILL) that supports clinicians in reviewing their practices and those of their peers and when demonstrating proportionality to outside reviewers [1].

### The use of coercion in somatic wards

Overall, the use of RP did not decrease in CDR during the study period; this cannot be explained by “boarding” in somatic emergency wards [47] as there are psychiatric emergency wards in all CDR psychiatric hospitals. We propose it might be due to the possible lag of training in de-escalation and specific treatment needs of psychiatric patients, as somatic wards did not participate in the implementation of Safewards. It is also possible that restrictive practices are used for very different conditions in somatic wards, such as delirium, organic and withdrawal states.

## Strengths and limitations

Results concerning the possible long-term Hawthorne effects are scarce, and only a few studies have demonstrated effects beyond six months [48]. Nevertheless, it cannot be ruled out that the decreasing use of RPs at the UH might be attributed to the Hawthorne effect.

RP and aggression prevention models in psychiatric wards are characterized by multifaceted and multidisciplinary approaches [4, 11, 49, 50]. The design features at the new UH aimed at reducing stress were implemented simultaneously. Furthermore, relocating and innovating an entire hospital has all the features of a complex intervention [51]. Consequently, this study can provide conclusions on individual measures such as ligature points, increased daylight and other measures.

Even though our study includes all admissions and restrictive practices from the self-contained CDR, which includes approximately 23% of the Danish population, our results can only be generalized to other countries with considerable caution due to the considerable international differences in legislation, clinical practices, and use of restrictive practices [52].

The UH (relocation group) and RHs (comparison group) were not entirely comparable, as the use of RPs at the RHs was lower (restrictive practices/admission) before the relocation compared to the UH. However, the UH is situated in a larger city and is obligated to treat the most complicated and treatment-resistant patients in Central Denmark Region, both of which might increase the actual use and decrease the possibilities of reducing RPs. Despite this, the UH succeeded in a continuous reduction of RPs during the study period as opposed to stagnation in the use of RPs at the RH. RH and UH are part of the same administrative organization and are governed by identical procedural and dynamical factors. Thus, the main difference between RH and UH during the study was the structural change of relocation at UH. Also, the UH and RH were comparable on clinical measures that may affect the use of RPs, such as readmissions, diagnostic admission patterns, and the proportion of patients with substance use disorder [53].

We collected data from a regional database for RP measures, which is continuously and thoroughly validated according to guidelines issued by The Danish Health Data Authority [54]. Furthermore, it is legally mandated to report all restrictive practices. Thus, selection bias and loss to follow-up are negligible.

## Implications for clinical practice and research

As several reviews have highlighted the relationship between the structural environment and health outcomes [6, 13, 55], clinicians need to be mindful of the effect of their physical surroundings in this vulnerable patient group [56]. Leaders need to be conscious of the impact of design decisions on treatment, therapeutic safety and security, and patient autonomy. Decisions on building or remodelling psychiatric facilities should be based on a complete framework [6]. This study's alignment between mechanical restraint and involuntary acute medication supports the proposal that restrictive practices can be reduced simultaneously without a substitution effect between the two [1].

Most models and studies that aim to reduce the use of RPs primarily focus on intramural factors, such as the culture in wards, the physical environment, training of staff, risk- assessment of inpatient violence, and the inclusion of patients in care decisions [4, 11]. However, a Danish study including 235 admitted patients found that RPs were predominantly prescribed during the very first hours of admission and that the risk of being subjected to RPs was significantly higher if a patient was involuntarily admitted (OR = 6.4 (3.4–11.9)), or were intoxicated by substances at admission (OR = 3.7 (1.7–8.2)). Thus extramural factors, such as outpatient treatment, accessibility to municipality services and substance abuse treatment, may impact the risk of being subjected to RPs once admitted [57]. Thus, our study indicates an untapped potential for the prevention of RPs as the proportion of RPs during the first 24 h of admission increased at both RH and UH during the study period.

This retrospective study aims to answer a narrow question and does not consider other possible impacts, nor does it consider how the new hospital interacted with the context in which it was implemented. To fully evaluate and understand the effects of a hospital relocation, we need to develop a theoretical framework, evidence-based indicators and methods [55].

## Conclusion

The naturalistic features of the design preclude any definitive conclusion whether relocation to a new purpose-built hospital decreased the use of RPs. However, we argue that improving the structural environment at the UH had a sustained effect on the already declining use of RPs, particularly mechanical restraint and involuntary medication.

## Data Availability

Data are not available.
